# Ptosis in childhood: A clinical sign of several disorders: Case series reports and literature review

**DOI:** 10.1097/MD.0000000000012124

**Published:** 2018-09-07

**Authors:** P. Pavone, Sung Yoon Cho, A.D. Praticò, R. Falsaperla, M. Ruggieri, Dong-Kyu Jin

**Affiliations:** a University-Hospital Policlinico-Vittorio Emanuele; b Department of Clinical and Experimental Medicine, Section of Pediatrics and Child Neuropsychiatry, University of Catania, Italy.; c Department of Pediatrics, Samsung Medical Center, Sungkyunkwan University School of Medicine, Seoul, Korea.

**Keywords:** ptosis

## Abstract

Blepharoptosis (ptosis) is a common but often overlooked sign that may serve as a sign/manifestation of other conditions, ranging from a mild and purely cosmetic presentation to a severe and occasionally progressive disorder. Ptosis may show an acute onset or may manifest as a chronic disorder. Its presentation may vary: unilateral versus bilateral, progressive versus non-progressive, isolated versus complex which occurs in association with other symptoms, and congenital versus acquired (often concomitant with neuromuscular disorders).

Congenital ptosis includes the isolated type—the congenital cranial dysinnervation disorders, which are further, distinguished into different subtypes such as Horner syndrome (HS), and ptosis as a sign/manifestation of various congenital malformation syndromes.

In this article, we review the primary causes of ptosis occurring in childhood, and its various clinical presentations, including a short report on selected cases observed in our institution: a classical isolated familial ptosis comprising 14 members over 5 generations, 3 sibling with isolated congenital ptosis who in addition suffered by episodes of febrile seizures, a patient with Duane retraction syndrome who presented congenital skin and hair anomalies, and a girl with HS who showed a history of congenital imperforate hymen. A flowchart outlining the congenital and acquired type of ptosis and the clinical approach to the management and treatment of children with this anomaly is reported.

## 1 Introduction

Blepharoptosis or ptosis, as it is more commonly known, is a common clinical sign that may affect individuals of all ages ranging from neonates to elderly individuals. Ptosis refers to a drooping or inferior displacement of the upper eyelid with associated narrowing of the vertical palpebral fissure. The drooping may be slight or insignificant; however, in a few patients, it might be severe in that the pupils are completely covered causing visual disturbances. This disorder is caused by a dysfunction of the muscles and/or nerves that regulate elevation of the eyelid.

**Figure 1:**
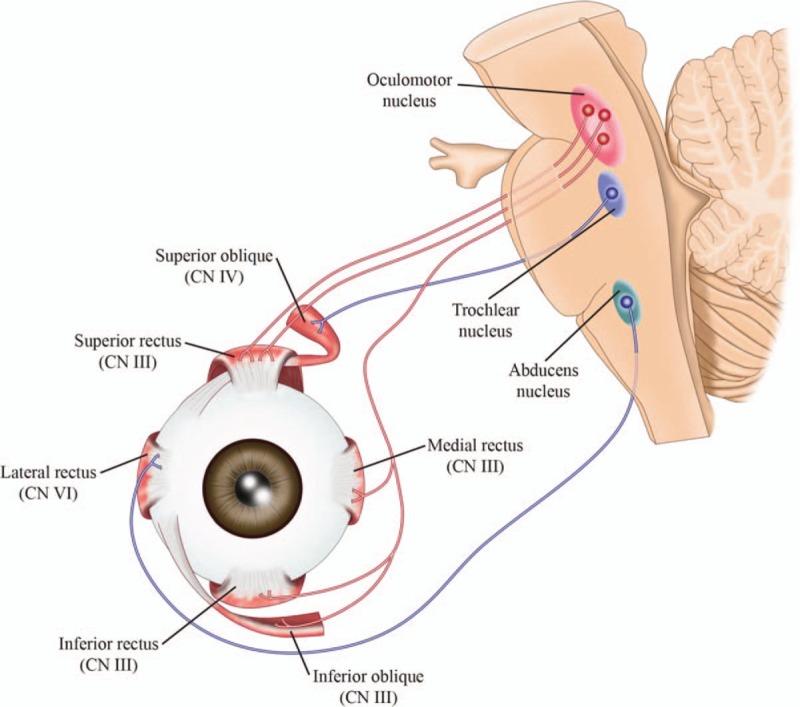
Representation of eyelid muscles and their innervation.

Two separate muscles are involved in the elevation of the eyelid—the levator palpebrae superioris, which is innervated by the superior branch of the III cranial nerve and the superior tarsal muscle (Müller's muscle), which is innervated by the cervical sympathetic system and elevates the posterior portion of the eyelid (Fig. 1).

**Figure 2:**
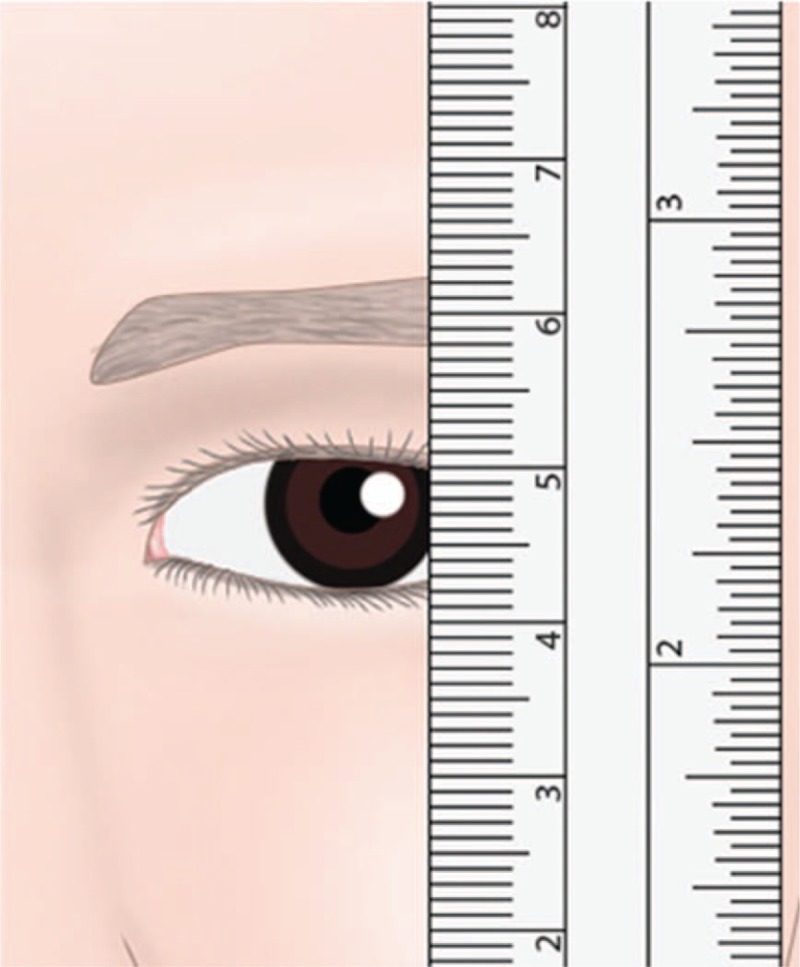
Measurement of the palpebral fissure height with a ruler.

Normally, the upper eyelid is positioned 1 to 2 mm below the upper corneal limbus, and the lower eyelid is placed at the sclerocorneal junction. Several methods are used to measure the inferior displacement of the upper eyelid. The most common approach in children is a measurement of the height of the palpebral fissure, which is defined as the widest distance between the upper and lower eyelid margins while the patient is in a primary gaze position (Fig. 2).^[1–3]^

**Figure 3:**
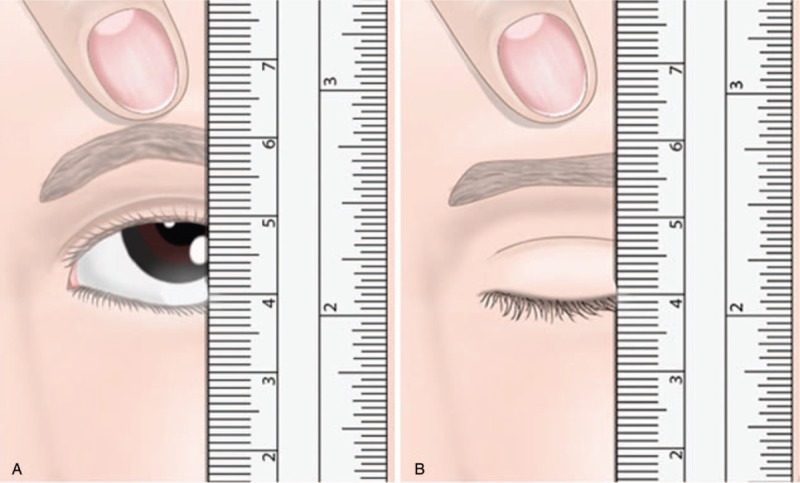
A&B. Eyelid measurement with a ruler using a pointing with a finger.

Other more sophisticated methods consist of measurement of the marginal reflex distance, upper eyelid creases, and the levator muscle function (Fig. 3 A and B).^[4,5]^

The incidence rate of ptosis is difficult to establish because of the diversity of disorders that are categorized within this group. The congenital type is the most common variety observed in childhood. Among the total number of patients referred for ptosis, Griepentrog et al observed congenital ptosis in 90% of 107 individuals,^[6]^ El Essawy et al observed ptosis in 68% of 236 children,^[7]^ and Berry-Brincat and Willshaw observed ptosis in 41% of 186 individuals (including 76 children).^[8]^ Furthermore, congenital ptosis was reported in 0.18% of 247,389 healthy individuals in a mass screening study performed by Hu.^[9]^

Ptosis may show several presentations. It may be familial or sporadic, acute or chronic, unilateral or bilateral, progressive or non-progressive, may manifest as an isolated sign or occur in association with other ocular anomalies and/or other systemic disorders of varying severity, and congenital (when noticed at birth or during the first year of life) or acquired often associated with neuromuscular disorders.

When examining a child with ptosis, it is important to distinguish between ptosis and “pseudoptosis,” which resembles the former condition; however, it occurs because of a different etiology. Pseudoptosis may be related to the lack of physical support to the eyelids secondary to a defective ocular globe, as is noted with some congenital ocular malformations, such as anophthalmia and microphthalmia.^[1]^ Dermatochalasis (redundant eyelid skin) may also cause pseudoptosis. Other known causes of pseudoptosis may be eye infections, corneal abrasions, or the presence of foreign bodies.

We review the primary causes of ptosis in childhood and present a short report on a few selected cases observed at our institution. All medical photos are presented with informed consent from patients and their parents. This study was approved by an autonomous Institutional review board of the Hospital Vittorio Emanuele of Catania.

Additionally, we discuss the clinical approaches to the management of ptosis and treatment of children affected by this anomaly.

## 2 Acute ptosis

Ptosis is a sign/manifestation of various disorders, and a few patients might present with an acute onset of this condition. Among these, Bell's palsy (facial nerve palsy) is the most typical example. It usually presents as an isolated entity, not associated with other cranial neuropathies or brainstem dysfunction. It results from a dysfunction of cranial nerve VII. The risk factors associated with Bell's palsy include diabetes and a recent upper respiratory tract infection. The presenting symptoms are unilateral facial weakness/paralysis of the facial muscles, with difficulty in closing or opening the eyelids associated with impaired taste and excessive tearing.^[2]^ Prednisone is used for treatment, which should be initiated at the onset of the condition, preferably within 72 hours.

Acute botulism is an infectious disease linked to the botulinum toxin, an exotoxin produced by the gram-positive bacterium *Clostridium botulinum*. Three primary clinical presentations of this entity are food-borne, infant, and wound botulism. Patients presenting with wound botulism develop neurological symptoms characterized by diplopia, blurred vision, ptosis, dry mouth, dysphagia, dysphonia, and dysarthria.^[10,11]^ This life-threatening disease requires administration of the human botulism immunoglobulin in the very early phases.

Miller–Fisher syndrome is considered a variant of Guillain–Barré syndrome and patients manifest with a classical triad of acute external ophthalmoplegia, ataxia, and areflexia. Similar to the presentation of Guillain–Barré syndrome, symptoms may be preceded by a viral illness. Treatment includes administration of intravenous immunoglobulins, steroids, plasmapheresis, and supportive care.^[12]^

Ophthalmoplegic migraine, which was previously regarded as a variant of migraine, has been recently considered a clinical expression of inflammatory cranial neuropathy,^[13]^ which may occur before, during, or after a migraine attack.

Third cranial nerve paralysis may be caused by traumatic, inflammatory, or neurotoxic events with ptosis being a primary symptom.

## 3 Chronic ptosis

Chronic ptosis is classified into the congenital and acquired varieties. Each type is further classified based on the etiological factors, as well as on isolated presentation or ptosis associated with other manifestations.

## 4 Congenital ptosis

### 4.1 Isolated congenital ptosis (ICP)

**Figure 4:**
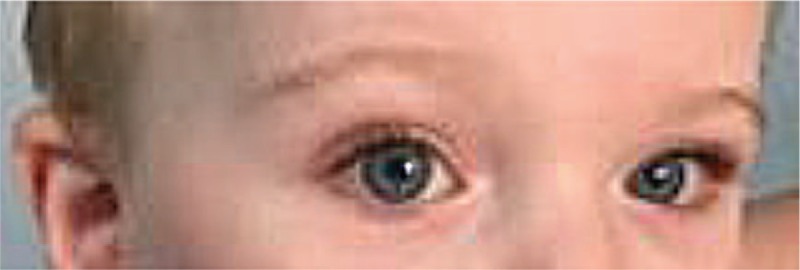
Image showing isolated congenital ptosis in a 2-year-old boy.

**Figure 5:**
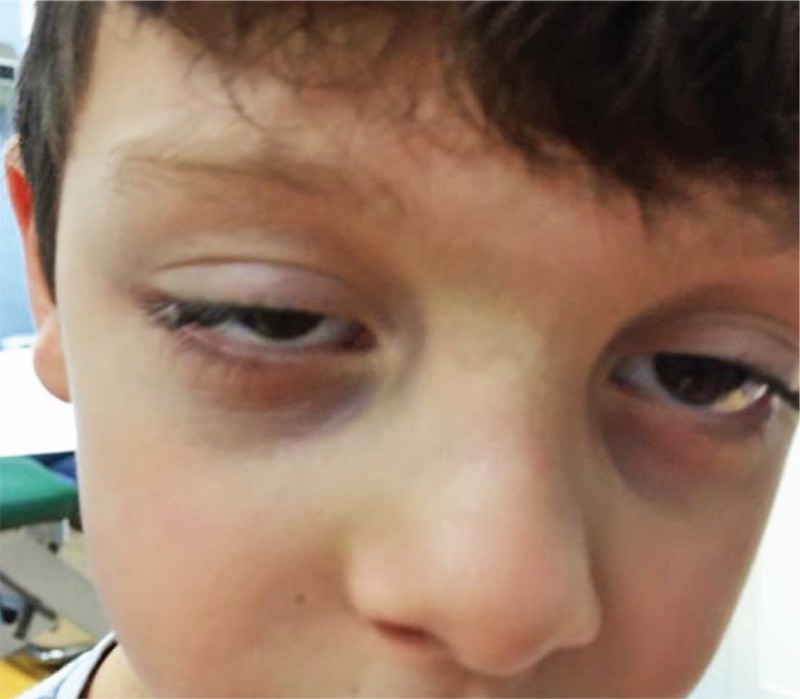
Image showing isolated congenital ptosis in a 12-year-old girl.

ICP is usually noticed in patients from birth; however, it may be observed within the first year of life. This anomaly tends to remain unmodified with time (Figs. 4 and 5).

Histopathological studies performed in individuals with ICP have shown an involvement of the levator palpebrae superioris muscle that demonstrates muscle fibrosis, fatty tissue infiltration, and a reduction in the number of muscle fibers.^[14,15]^ More recently, however, a neurological etiology has been considered for this anomaly with this condition being attributed to defective/abnormal muscle innervation.^[16]^

A study performed by Engle et al^[17]^ showed that in 42 members of a family in whom 20 individuals were affected by ptosis, a 3-cM region, which was assigned the PTOS1 loci, located on the 1p32−34.1 chromosome was identified as being responsible for ptosis. McMullan et al^[18]^ performed genetic linkage studies in a large family with an X-linked dominant congenital condition that revealed a critical region located between Xp24 and Xq27.1.^[18]^ Additionally, McMullan et al^[19]^ identified the *ZFH4* gene, located on 8q21.12 as a candidate gene for ICP in a child with bilateral ICP, with a balanced translocation of chromosomes 8 and 10. A mutation in chromosome 8 disrupts the *ZFH4* gene, which encodes a protein with a zinc-finger homeodomain that acts as a transcription factor. This protein interferes with normal muscle and nerve development causing dysfunction of the cranial nerves and the muscles they innervate.^[19,20]^ Nakashima et al^[21]^ proposed 3 candidate disease-responsible regions, 8q21.11−q22.1, 12q24.32−33, and 14q21.1−q23.2 for PTOS1, utilizing whole-genome linkage analysis performed in a Japanese family.^[21]^

Upon clinical examination, patients with ICP may show symmetric or asymmetric, and unilateral or bilateral involvement, with more frequent involvement of the left eyelid (approximately two-thirds of all cases reported).^[2]^ A retrospective study performed by Griepentrog et al^[6]^ showed that ICP was observed in 1 of 842 births, and the left eyelid was affected in 55% of the patients studied. Pavone et al reported a study involving a family comprising 14 members over 5 generations demonstrating ICP with an autosomal dominant pattern of inheritance and 70% to 90% penetrance.^[22]^

We have followed up this family for about 15 years. In this family, the affected individuals showed both unilateral and bilateral involvements with some of the family members presenting also synkinesia. The ptosis remained unchanged in all the individuals along this period of time. In the majority of the cases, the left eyelid was the most affected side.

Here we report on 3 siblings (2 brothers and a sister who showed ICP and all suffering in childhood by frequent episodes of febrile seizures (FS). A 5 years old boy came to our observation because of episodes of febrile seizures. He was the first child of unrelated Italian parents. The family history disclosed the presence of ICP involving the left side in the paternal line and childhood episodes of febrile seizures in the mother line. The child was born at 36 weeks of gestation by cesarean section. His birth weight was 3200 g, his height 49 cm and head circumference 36 cm, all within the normal range. Soon after birth, unilateral left side ptosis was noted. His developmental milestones were reached normally. Since the years of 2 years, the child suffered from frequent episodes of tonic-clonic generalized seizures in association with high fever lasting few minutes and not associated to postictal neurological involvement. The electroencephalography (EEG) was normal. During the previous 3 years the child presented with several episodes of the FS with frequency of 3 to 4 episodes for years. At the age of 3 years due to the high frequency of FS episodes treatment with valproate at the dosage of 20 mg/kg-day was started, but the seizures were still reported in most of the febrile episodes. Neurologic examination and psychiatric evaluation, as well as heart, thorax, abdomen, and general organs were assessed as normal, and the growth parameters were within the normal range. Routine laboratory analysis and EEG were normal while awake and during the sleep in various admissions to the hospital. The child and his siblings affected by ICP suffered in childhood by FS which were inherited from the maternal line whereas the ptosis was inherited from the paternal side. The FS episodes in the child and in his siblings disappeared from the age of 5 years.

In a case series comprising of 60 patients studied over a 5-year period, 8 patients (13%) were observed to have been affected by ICP (23) (unpublished report).

**Figure 6:**
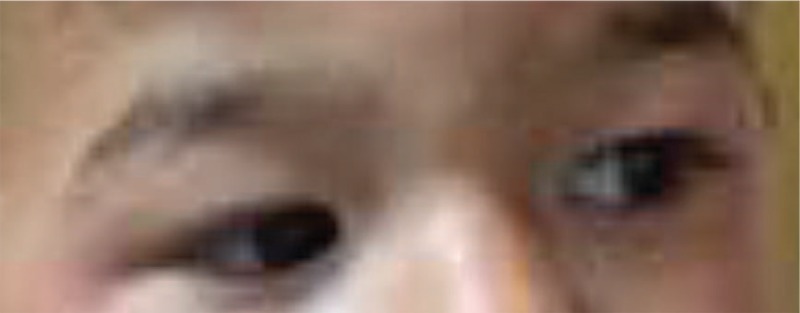
Image showing a 4-year-old boy with ptosis. Isolated congenital ptosis was also observed in his sister and brother. All 3 children presented with ptosis and episodes of febrile seizures.

In a case series comprising 60 patients studied over a 5-year period, 8 patients (13%) were observed to have been affected by ICP.^[23]^ (unpublished report) (Fig. 6).

ICP treatment depends upon the severity of the anomaly following evaluation of the margin-reflex distance, levator excursion test, presence of amblyopia, and based on the decision of the parents and family. Surgical repair of severe ptosis is performed after 6 months of age in agreement with the family's decision. Although surgical treatment involves the use of several techniques, the most commonly recommended treatment involves anterior levator aponeurotic muscle resection or a frontalis sling or suspension procedure.^[24]^

### 4.2 Congenital cranial dysinnervation disorders (CCDDs)

CCDDs encompass a group of disorders resulting from anomalous innervation of the ocular and facial musculature.^[25]^ These disorders include Duane retraction syndrome (DRS), blepharophimosis ptosis epicanthus inversus syndrome (BPES), congenital fibrosis of the extraocular muscles (CFEOM), and the Marcus Gunn phenomenon.

### 4.3 DRS

Patients diagnosed with DRS may present with abduction paresis (type 1, most common), adduction paresis (type 2, least common) or both (type 3, second most common). Contraction of the lateral rectus muscle upon attempted adduction causes retraction of the ocular globe and subsequent enophthalmos and ptosis. The 5 cardinal features of DRS type I include congenital onset, severely limited abduction, slightly limited adduction, retraction of the globe and palpebral fissure narrowing, as well as up-shoot and down-shoot on adduction. Cytogenetic abnormalities, on both, the long arm of chromosome 2 (2q31) and chromosome 8 (8q13) have been reported.^[26]^

**Figure 7:**
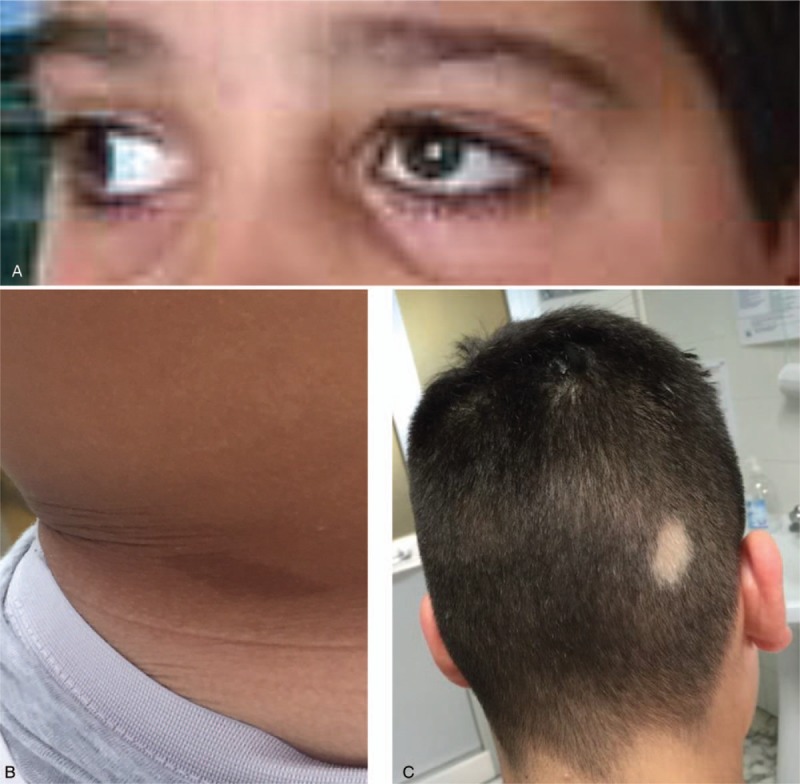
Image showing a 10-year-old boy with Duane retraction syndrome. Ptosis (A) is observed to be associated with café-au-lait spots in the trunk (B), and piebaldism (C).

We recently observed a 10-year-old boy affected by DRS type 1 who presented in association cutaneous and hair anomalies consisting of 3 to 4 café-au-lait spots measuring 3 to 5 cm in size localized to the trunk and a small patch of piebaldism on the scalp (Fig. 7 A–C). The boy came to our observation at the age of 3 years and 7 months for psychomotor delay and dysmorphic features. He is the third child of healthy unrelated parents. The 2 older siblings were born from the former maternal marriage and are healthy. At the time of the conception, the mother was 28 years old and the father 31 years old. During the proband's pregnancy, the mother denied having had infections or gestosis or consuming alcohol or drugs. She did not take supplementary folic acid.

The boy was born at 39th week of gestation from normal delivery. The birth-weight was 3.3 Kg, length 50 cm, and head circumference 35 cm. The proband since his first months of life showed a delay in the development milestones. The first convulsive episode started at the age of 5 months, with a tonic-clonic crisis lasting a few minutes. After this episode, other seizures occurred with a frequency of 2 to 3 each month. Treatment with valproate at normal dosage yielded a noticeable reduction of the epileptic seizures. At the age of 5 years and 7 months, at the physical examination, general conditions were good. His weight was 19 Kg (50th percentile), height 105 cm (10–25th percentile) and head circumference 51 cm (50th percentile). He shows minor facial dysmorphisms consisting in large forehead with protruding metopic suture, bilateral ptosis, more evident in the left eye, hyperplasia of the eyelashes, mildly convergent in the centrum. At the neurological examination, the muscular tone and strength was normal, as was the cranial nerves, and the patellar reflexes. Hearth, thorax, internal organs were normal. Routine laboratory analyses were normal.

Results of genetic testing micro-array showed the following re-arrangement: arr[hg19] 22q11.22 (22.859.095–23.353.058 × 3). Same rearrangement was found in the father whereas the result in the mother was negative. presented also in the father. In the mother, array-CGH analysis was negative.

### 4.4 Blepharophimosis ptosis epicanthus inversus syndrome (BPES)

In addition to ptosis, telecanthus, and premature ovarian failure in women may be associated with BPES. In contrast to type 2 BPES, premature ovarian failure is observed only in the type 1 variant. This condition has been attributed to a mutation in the transcription factor FOXL2, resulting in the production of truncated proteins in the mesenchyme of the developing eyelid structure.^[27]^

### 4.5 Congenital fibrosis of the extraocular muscles (CFEOM)

CFEOM includes a group of disorders with non-progressive restrictive ophthalmoplegia of the extraocular muscles, which are innervated by the oculomotor, trochlear, and abducens nerves manifesting with congenital blepharoptosis. Three clinical phenotypes have been distinguished. Type 1 is inherited in an autosomal dominant fashion, and the responsible gene is *KIF21A*, located on chromosome 12p11.2–q12, which encodes an anterograde kinesin motor protein.^[28–30]^ The position of both eyes is below the horizontal midline with severe restriction of elevation of either eye. Type 2 CFEOM is inherited in an autosomal recessive fashion, and the responsible gene is *POX2A/ARIX*, located on chromosome 11q13.1.^[31]^ Type 3 CFEOM is inherited in an autosomal dominant fashion and is caused by a mutation in *TUBB3* and *KIF21A* genes. This type is characterized by congenital bilateral exotropic ophthalmoplegia and ptosis, with pupillary abnormalities and manifests with ptosis, and ophthalmoplegia affecting the vertically acting extraocular muscles. This type could be associated with intellectual and behavioral impairment in a few patients.^[30,32–34]^

### 4.6 Marcus Gunn (MG) jaw-winking syndrome

MG jaw-winking syndrome is caused by a synkinetic anomaly secondary to aberrant innervation of the mandibular branch of the trigeminal nerve innervating the levator palpebrae muscle. Following the contraction of the pterygoid muscle, the ptotic eyelid is elevated and retracts, causing a winking movement during eating, chewing, or sucking actions. No pathogenic cause has been identified for this anomaly.^[35–37]^

### 4.7 Horner syndrome (HS)

HS results from a disruption in the sympathetic nervous system pathway extending between the brain and the Müller's muscle, thereby affecting the eye and ipsilateral side of the face. Patients with HS manifest in ipsilateral ptosis, miosis, and anhidrosis. The disorder may be congenital and could be associated with a lighter color of the iris of the affected eye. HS may present in association with contralateral hemifacial flushing and ipsilateral hypohidrosis (Harlequin syndrome). Dystocic delivery, often associated with brachial plexus injury, is considered a possible cause of HS.^[38,39]^.

Recently, we observed a young girl with HS (miosis and mild ptosis in the absence of anhidrosis) who presented with a history of congenital imperforate hymen. This 4 years old girl was admitted as outpatient to our institution with the diagnoses of HS. The neonatal history displayed a surgical intervention for imperforate hymen with the absence of a vaginal opening and bulging of vagina. The subsequent period and the developmental stages were normal. The anomaly was initially overlooked, at the age of 3 years old due to the presence of miosis, partial ptosis, and mild hypohydrosis the diagnosis of congenital HS was performed. Physical and laboratory examination and neuroimaging were normal. Cocaine test confirmed the diagnosis of HS. At the present age of 8 years old the girls leads a normal life and good scholastic performances.

Although HS may also be an acquired entity, this variant is less commonly reported in children compared with congenital HS. The disorder may appear as a consequence of cerebral lesions, with lesions located between the hypothalamus and the fibers moving from the spinal cord or a peripheral location in the superior cervical ganglia or cervical sympathetic chain. Tumors, neuromyelitis optica, postviral damage, internal carotid artery agenesis, cervical disc herniation, as well as neuroblastomas and other associated disorders may be the possible etiological factors contributing to the development of this disease entity.^[38–40]^

### 4.8 Congenital facial palsy (CFP)

CFP is usually traumatic and rarely developmental in origin. In the latter case, patients may present with microtia and atresia of the external auditory canals as anomalies associated with the disorder.^[2,3]^

### 4.9 Congenital myasthenic syndromes (CMS)

CMS are a heterogeneous group of disorders affecting neuromuscular transmission. These disorders are distinguished by molecular defects and the localization of the dysfunction at the presynaptic, postsynaptic, and neuromuscular junction. Mild ptosis is the most common sign in patients, although the severity and course of the disorder could vary, involving ocular or bulbar and limb muscle impairment. Patients may present in the neonatal period with respiratory failure with episodes of apnea, cyanosis, and generalized weakness. The genes most commonly associated with this condition are *CHAT*, *CHRNE*, *COLQ*, *DOK7*, *GFPT1,* and *RAPSN*.^[41–43]^ Treatment consists of the administration of acetylcholinesterase inhibitors and/or 3,4-diaminopyridine, a potent potassium channel blocker.

### 4.10 Congenital ptosis in congenital malformation syndromes

#### 4.10.1 Turner syndrome (TS)

This syndrome is the most common sex chromosome abnormality observed in females, with an incidence of 1 in 2500 live female births. The primary features of TS consist of growth retardation, gonadal dysgenesis, congenital and acquired cardiovascular anomalies, and a specific cognitive and psychosocial phenotype. Other signs observed in patients include a webbed neck, shield chest, epicanthal folds, and ptosis. The presence of edematous hands and feet, related to lymphedema in a newborn are suggestive of the diagnosis. The classic phenotypic presentation is associated with the 45, X karyotype.^[44,45]^

#### 4.10.2 Noonan syndrome (NS)

NS is an autosomal dominant disorder in which patients show a broad spectrum of clinical presentations including unusual facial features, congenital heart abnormalities, increased rate of tumor incidence, a webbed neck, as well as characteristic chest anomalies such as superior pectus carinatum and inferior pectus excavatum. Other characteristic features/signs are related to coagulation defects, lymphatic dysplasia, and ocular anomalies including ptosis and hypertelorism. NS is the result of germline mutations in the genes that encode protein components of the intracellular RAS/MAPK pathway.^[46]^

#### 4.10.3 Smith–Lemli–Opitz syndrome (SLOS)

SLOS is one of the multiple congenital malformation syndromes showing an autosomal recessive pattern of inheritance. It occurs secondary to an inborn error of cholesterol metabolism linked to a deficiency of the enzyme 7-dehydrocholesterol reductase. Patients with SLOS type 1 present with bitemporal narrowing, growth retardation, pre- and postnatal microcephaly, and moderate-to-severe intellectual disability. Facial and systemic malformations include ptosis, cleft palate, cardiac defects, underdeveloped external genitalia, post-axial polydactyly, and 2 to 3 toe syndactyly.^[47]^

#### 4.10.4 Rubinstein–Taybi syndrome (RSTS)

RSTS is a congenital malformation in which patients present with distinctive facial features, broad and short thumbs, and first toes. The latter feature is considered typically suggestive of the diagnosis. The craniofacial dysmorphism in patients diagnosed with this condition consists of microcephaly, a low anterior hair line, downslanting palpebral fissures, ocular signs (including ptosis, epicanthus, and strabismus), a broad nasal bridge, a beaked nose, and a prominent columella. Mutations in 2 functionally related genes, *CREBBP* and *EP300*, have been reported as the causative factors in 55% to 78% of patients.^[48,49]^

## 5 Acquired ptosis

As is noted with the congenital variety, ptosis is a sign/manifestation of various acquired disorders. However, in contrast to the congenital form, acquired ptosis is usually characterized by a progressive and severe/serious course. Several metabolic, neuromuscular, muscular, and/or traumatic conditions may clinically present with ptosis.

### 5.1 Chronic progressive external ophthalmoplegia (CPEO)

CPEO is a mitochondrial disease with gradually progressive, usually bilateral ptosis, and limited ocular mobility in all directions of gaze.^[50,51]^ Mitochondria are ubiquitously distributed cellular organelles; therefore, mutations in mitochondrial DNA or associated nuclear mutations present with a vast spectrum of systemic disorders. CPEO encompasses a heterogeneous and wide-ranging group of disorders including the above-mentioned CPEO syndrome in which patients might show mild clinical features restricted to bilateral ptosis.^[51,52]^ Other examples are the Kearns–Sayre syndrome, in which patients manifest with progressive external ophthalmoplegia, retinitis pigmentosa, and heart blocks. Cerebellar ataxia, sensorineural hearing loss, and increased protein content of the cerebrospinal fluid have also been reported. Muscle biopsy evaluation and molecular genetic analysis may help to confirm the diagnosis. Histopathological analysis of muscle tissue shows ragged-red, and ragged-blue fibers, or cytochrome C oxidase-negative fibers.^[53]^

Patients diagnosed with Pearson marrow-pancreas syndrome present with sideroblastic anemia with vacuolization of marrow precursors and exocrine pancreatic dysfunction.^[54]^ Several other diseases may present with CPEO such as mitochondrial encephalomyopathy lactic acidosis and stroke-like episodes (MELAS), mitochondrial neurogastrointestinal encephalomyopathy (MNGIE) caused by mutations in the nuclear gene *TYMP*, as well as sensory ataxic neuropathy, dysarthria/dysphagia, and external ophthalmoplegia (SANDO).^[55–57]^ Other disorders such as spinocerebellar degeneration, Refsum's disease, and abetalipoproteinemia may also be included in the CPEO category of disorders.^[1]^

### 5.2 Oculopharyngeal muscular dystrophy (OPMD)

OPMD is an autosomal dominant disorder in which patients present with ptosis, swallowing anomalies, proximal limb weakness, and presence of nuclear aggregates in muscles. The disorder is caused by a trinucleotide repeat expansion in the *PABPN1* gene. Presently animal research is underway to identify an optimal treatment strategy for patients with OPMD.^[58]^

### 5.3 Myotonic dystrophy (dystrophia myotonica [DM])

DM is a genetic disorder showing an autosomal dominant pattern of inheritance that affects muscles and other organs including the heart and the brain. The primary clinical features observed in patients include progressive muscular weakness, atrophy, and myotonia in addition to ophthalmologic involvement in the form of ptosis, cataracts, and pigmentary retinopathy. The disorder runs a progressive course with patients showing accelerated aging and rapidly worsening muscular weakness, cognitive decline, and metabolic dysfunction. Myotonia is exacerbated by fatigue, cold, and excitement. Two types of DM have been recognized—type 1 is caused by an expansion of a CTG triplet repeat in the *DMPK* gene, whereas type 2 is caused by an expansion of a CCTG tetramer repeat in *CNBP*. DM mutations lead to the expression of dominant-acting RNAs in all patients.^[59,60]^

Diagnostic tests used are: progressively worsening ptosis with sustained upward gaze for 1 or 2 minutes, and rapid opening and closing of fists causes fatigue of the hand muscles and difficulty in elevation of the arms for greater than 1 to 2 minutes. The ice and the Tensilon test demonstrate good sensitivity in diagnosing this condition. Administration of Tensilon (a short-acting acetylcholinesterase inhibitor) demonstrates an improvement in ptosis and ophthalmoplegia. Treatment with acetylcholinesterase inhibitors is widely utilized.^[2]^

### 5.4 Myasthenia gravis

Acquired autoimmune myasthenia gravis (AMG) is a postsynaptic neuromuscular disorder in which patients present with heterogeneous clinical signs and the presence of antibodies. These antibodies are directed against a component of the neuromuscular junction, most commonly, the acetylcholine receptors.^[61]^ Reportedly, an antibody directed against the nicotinic acetylcholine receptor has been demonstrated in 85% of the patients diagnosed with generalized AMG and 50% of patients with ocular myasthenia gravis (OMG). Patients with OMG present with a history of painless weakness or fatigability of the extraocular muscles, and ptosis. A progressive decrement in the muscle action potential response to repetitive nerve stimulation at 2 to 3 Hz confirms the diagnosis of this disorder. In patients showing a negative response, diagnosis may be confirmed through single fiber electromyography. Treatment with acetylcholinesterase inhibitors may show a good effect in managing ptosis.^[62,63]^

## 6 Surgical treatment of ptosis

The primary objective of surgery in the management of ptosis is to restore normal eyelid functions, essentially by elevating the position of 1 or both eyelids and by creating a lid fold, if necessary. Moreover, special attention is needed for maintenance of proper contour and symmetry of the lids.

Patients or their caregivers should be aware of the several limitations associated with this condition and that the results may not be conclusive—particularly in those with congenital ptosis because factors inherent to the anatomic defect can limit the surgical results. The expectations and goals of the surgery for children being performed must be discussed carefully with either the parents and caregivers or both, preoperatively. A defective levator muscle showing abnormal and/or absent function preoperatively, cannot be restored surgically. The lid level can be changed through a modified Crawford technique, although dynamic limitations in the affected muscle are known to persist postoperatively, causing significant lid lag or lagophthalmos. Other complications may be inappropriate eyelid closure, an exacerbation of a pre-existing tear deficiency, and secondary exposure keratopathy.^[64]^

## 7 Conclusions

**Figure 8:**
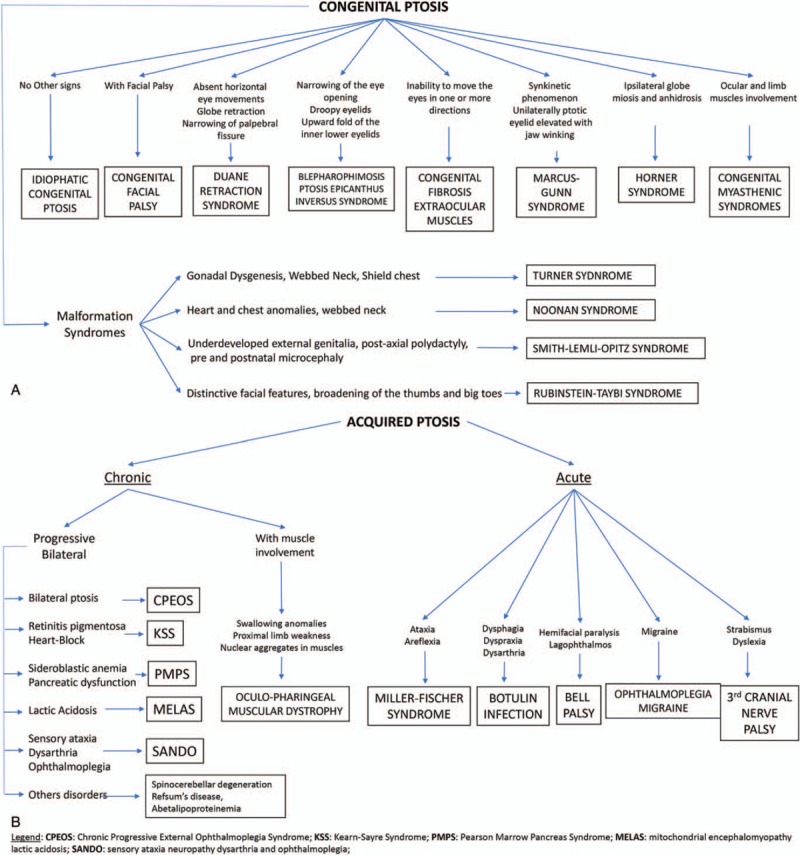
Flow chart outlining ptosis: congenital (A) and acquired (B) types.

In conclusion, ptosis is a noticeable sign associated with various diseases, ranging from a mild to a very severe type, with the involvement of various body organs. Usually, ptosis can be easily identified clinically; however, diagnosis might be difficult in patients presenting with complex types that are associated with diverse manifestations. A flowchart outlining the clinical diagnosis of ptosis has been summarized in Figure 8. Although a few of these disorders are amenable to treatment, there is no definitive therapy available for many of these conditions.

## Acknowledgments

We thank all the individuals who have been diagnosed with the afore-mentioned rare diseases and their families and all the clinical and research laboratory staff who have been instrumental in the successful completion of our work. This study was supported by a grant from Samsung Medical Center (#GFO2170061).

## Author contributions

**Data curation and flow chart compilation:** AD Praticò.

**Formal analysis:** AD Praticò, R Falsaperla.

**Methodology:** M Ruggieri.

**Supervision:** Dong-Kyu Jin.

**Validation:** R Falsaperla.

**Visualization:** M Ruggieri.

**Writing – original draft:** P Pavone.

**Writing – review & editing:** Sung Yoon Cho.
